# The interplay between autophagy and cGAS-STING signaling and its implications for cancer

**DOI:** 10.3389/fimmu.2024.1356369

**Published:** 2024-04-10

**Authors:** Maximilian Schmid, Patrick Fischer, Magdalena Engl, Joachim Widder, Sylvia Kerschbaum-Gruber, Dea Slade

**Affiliations:** ^1^ Department of Radiation Oncology, Medical University of Vienna, Vienna, Austria; ^2^ Comprehensive Cancer Center, Medical University of Vienna, Vienna, Austria; ^3^ MedAustron Ion Therapy Center, Wiener Neustadt, Austria; ^4^ Department of Medical Biochemistry, Medical University of Vienna, Max Perutz Labs, Vienna Biocenter, Vienna, Austria; ^5^ Vienna Biocenter PhD Program, a Doctoral School of the University of Vienna and Medical University of Vienna, Vienna, Austria

**Keywords:** autophagy, cGAS/STING signaling, innate immunity, cancer, radiotherapy

## Abstract

Autophagy is an intracellular process that targets various cargos for degradation, including members of the cGAS-STING signaling cascade. cGAS-STING senses cytosolic double-stranded DNA and triggers an innate immune response through type I interferons. Emerging evidence suggests that autophagy plays a crucial role in regulating and fine-tuning cGAS-STING signaling. Reciprocally, cGAS-STING pathway members can actively induce canonical as well as various non-canonical forms of autophagy, establishing a regulatory network of feedback mechanisms that alter both the cGAS-STING and the autophagic pathway. The crosstalk between autophagy and the cGAS-STING pathway impacts a wide variety of cellular processes such as protection against pathogenic infections as well as signaling in neurodegenerative disease, autoinflammatory disease and cancer. Here we provide a comprehensive overview of the mechanisms involved in autophagy and cGAS-STING signaling, with a specific focus on the interactions between the two pathways and their importance for cancer.

## 1 Autophagy

Autophagy is an adaptive, highly conserved cellular process that acts as a recycling mechanism to maintain cellular homeostasis. It has long been thought of as an unspecific degradation system to regenerate nutrients. However, in recent years autophagy emerged as a selective process that degrades potentially dangerous cellular materials ranging from misfolded proteins and protein aggregates to damaged mitochondria and intracellular bacteria (1–3). Autophagy can also be used to secrete cytosolic proteins (termed secretory autophagy) and can regulate immune responses by secreting damage-associated molecular patterns (DAMPs) or cytokines (4–7). There are three main types of digestive autophagy in mammalian cells: macroautophagy, microautophagy and chaperon-mediated autophagy. They employ different mechanisms to converge at the same final step of cargo delivery to lysosomes followed by its proteolytic degradation. In this review we will only focus on macroautophagy and will, for simplicity, refer to it as “autophagy”. In brief, the process of macroautophagy involves the formation of double membrane vesicles called autophagosomes that form around cellular cargo and travel to lysosomes where they subsequently fuse with them to induce cargo degradation (1, 8, 9). Autophagy is induced by different forms of cellular stress and is thereby connected to the main stress signaling pathways of the cell: nutrient availability [mTOR (10, 11)], energy status [AMPK (12, 13)], hypoxia [HIF (14)] and infection [inflammation (15)]. As a consequence, autophagy is also implicated in major pathologies like neurodegenerative disease, pathogenic infections and cancer, where it can either act in a cytoprotective or cytotoxic fashion and can even accelerate the course of the disease (1, 2, 16–19). Targeting autophagy therefore represents a promising strategy against these pathologies.

### 1.1 The mechanism of autophagy

Generally, autophagy consists of four distinct phases: (1) initiation, (2) nucleation, (3) elongation, (4) fusion and degradation, which involve many protein complexes required for autophagosome biogenesis, trafficking, fusion with lysosomes, and cargo degradation (1, 20–22).

#### 1.1.1 Initiation

During the initiation of autophagy, the Unc-51-like kinase (ULK) complex, consisting of either ULK1 or ULK2 (a serine/threonine kinase), RB1-inducible coiled-coil protein 1 (FIP200), autophagy related protein 13 (ATG13) and autophagy related protein 101 (ATG101), induces the formation of autophagosomes by acting as a scaffold to recruit autophagic machinery (1, 20, 22). ULK1 directly interacts with the mammalian target of rapamycin complex I (mTORC1) and AMP-activated protein kinase (AMPK) via phosphorylation (23, 24). While autophagy inhibition by mTORC1 and induction by AMPK are the most common routes, there are many more pathways that can activate autophagy. These range from endoplasmic reticulum (ER) stress to hypoxia, microbial infection, DNA damage and mechanical stress (14, 25–32). Some of these will be mentioned in more detail in later parts of this review.

**Figure 1:**
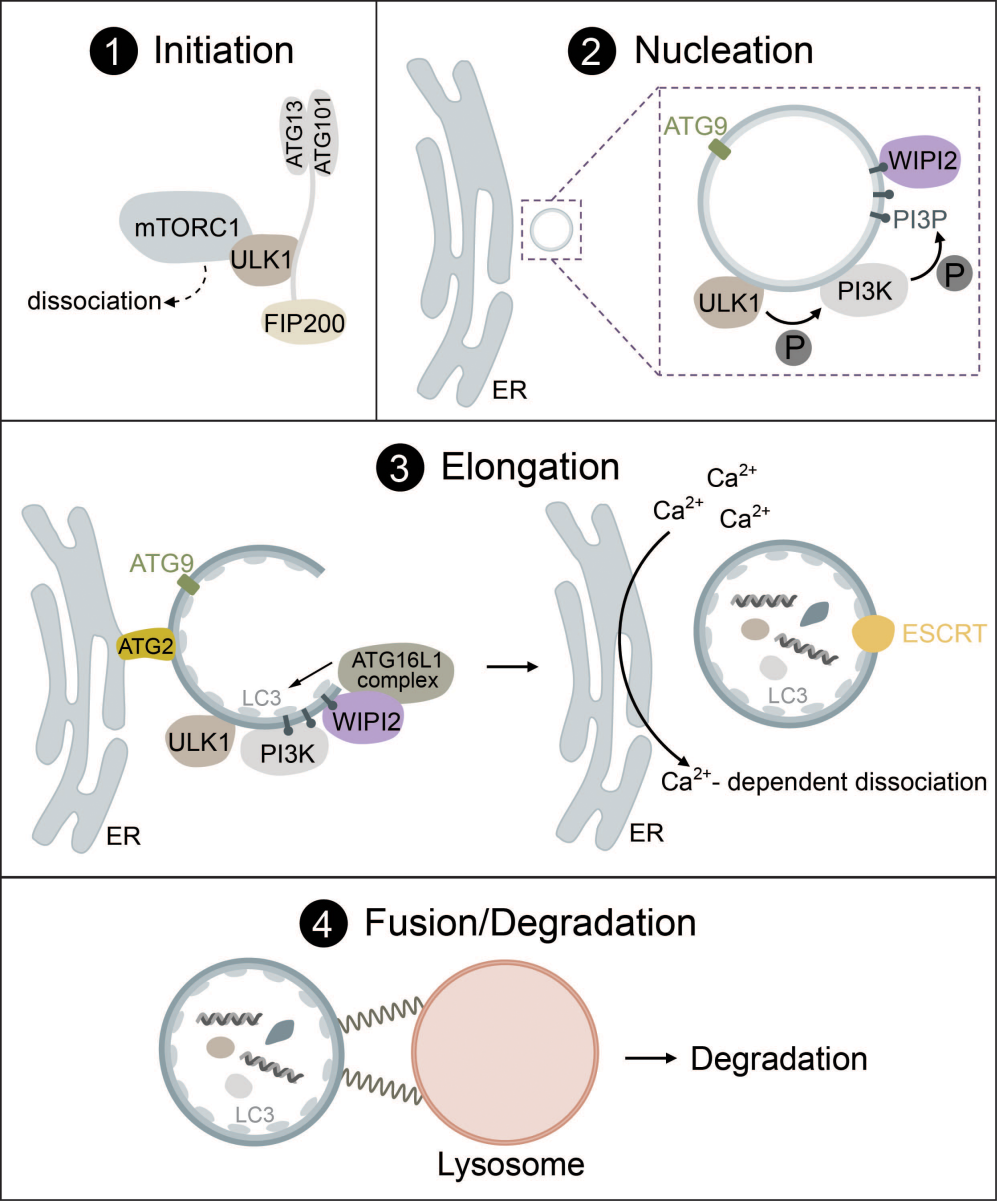
The mechanism of autophagosome biogenesis. 1) Upon starvation, mTORC1 dissociates from ULK1, which is then activated via autophosphorylation and phosphorylates ATG13 and FIP200. 2) ULK1 is recruited to the phagophore assembly site together with ATG9-positive vesicles. ULK1 phosphorylates the PI3K complex, inducing the production of PI3P. PI3P leads to the recruitment of WIPI2. 3) The ATG16L1 complex is recruited to the growing autophagosome via WIPI2 and conjugates ATG8-family proteins such as LC3 to PE. Lipid influx is controlled via ATG2, which acts as a channel funneling lipids from donor compartments. Autophagosome closure is mediated by the ESCRT complex. Membrane contacts are dissolved via VMP1-mediated changes in local Ca^2+^ concentration. 4) Fully formed autophagosomes travel to the lysosome and undergo fusion mediated by SNARE proteins, tethering factors and RABs. Cargo is then degraded by lysosomal hydrolases.

Under high nutrient availability, mTORC1 is activated and negatively regulates the ULK complex by directly binding and inactivating ULK1 and ATG13 by phosphorylation. Upon starvation, however, mTORC1 is deactivated and ULK1 is dephosphorylated, causing it to dissociate from mTORC1 (**Figure 1**, panel 1). Simultaneously, ULK1 is activated by autophosphorylation, which triggers phosphorylation of its substrates ATG13 and FIP200 (24, 33, 34). ULK1 then forms multimeric complexes that act as a recruitment platform for downstream autophagy proteins such as the phosphoinositol 3-kinase (PI3K) complex I as well as multiple adaptor proteins that are essential for autophagosome biogenesis (35–37).

Another regulator of autophagy is the energy-sensing protein kinase AMPK. Cellular starvation caused by, for example, low levels of glucose results in an increased AMP/ATP ratio, which activates AMPK. Activated AMPK can initiate autophagy indirectly by inactivating mTORC1 or directly by phosphorylating ULK1 (24, 38, 39).

Autophagy regulation by both mTORC1 and AMPK is, however, generally very unspecific and only responds to certain nutrient conditions such as glucose or amino acid starvation (24). Selective autophagy follows a similar initiation procedure that relies on the assembly of multiple ULK complexes but is mostly independent of mTORC1 (40, 41). These ULK1 assemblies form on cargo marked by specific autophagic cargo receptors, such as sequestosome-1 (SQSTM1/p62) and nuclear domain 10 protein 52 (NDP52) (42–44).

#### 1.1.2 Nucleation

The nucleation phase of autophagy has proven to be exceedingly complicated and hard to study, which is why it is still not fully understood. In the following section we will outline the main steps based on the current state of knowledge.

While various organelles have been shown to contribute to autophagosome biogenesis, it has become clear that the ER serves as a platform for this process in most cases (45–47). During nucleation, the ULK1 complex is recruited to the phagophore assembly site (PAS) at ER regions, which are enriched in vacuole membrane protein 1 (VMP1) (48–50). ER transmembrane proteins VAPA/B facilitate the recruitment of the ULK1 complex through direct interaction with FIP200 and ULK1 (51). ATG9, a transmembrane protein found in vesicles that originate from the trans-Golgi, endosomes or even the plasma membrane, is recruited to the PAS together with ULK1 and has been implicated as a seed for the initial establishment of membrane contact sites in yeast (52) and mammals (53, 54) (**Figure 1**, panel 2).

In the next step, ULK1 initiates the synthesis of phosphatidylinositol 3-phosphate (PI3P) via the PI3K complex I that consists of vacuolar sorting protein 34 (VPS34), 150kDa protein (p150), Bcl-2 interacting protein (Beclin-1), ATG14L and nuclear receptor-binding factor 2 (NRBF2). ULK1 directly phosphorylates Beclin-1, which leads to the activation of VPS34 kinase activity that produces PI3P (55–59). PI3P then regulates the recruitment of several downstream autophagic factors such as WD repeat domain phosphoinositide-interacting protein 2 (WIPI2) or the PI3P-binding protein zinc-finger FYVE domain-containing protein 1 (DFCP1) (49, 60, 61). These PI3P-rich, DFCP1-positive regions are termed the omegasome and are distinct from the autophagosome or pre-autophagosome. Omegasomes act as a platform for the transformation of phagophores into fully-fledged autophagosomes as well as for membrane expansion and budding of fully-formed autophagosomes (49, 62–64). WIPI proteins serve as PI3P effectors and function as an adaptor for the lipidation machinery (ATG16L1 complex) driving membrane expansion in a PI3P-dependent manner (60, 65) (**Figure 1**, panel 2).

#### 1.1.3 Elongation

Elongation of the phagophore requires the interplay of two complexes that function as ubiquitin-like systems: the ATG16L1 complex and the ATG8 lipidation machinery. The formation of the ATG16L1 complex is initiated by activation of the ubiquitin-like protein ATG12 by ATG7 (E1-like enzyme) in an ATP-dependent manner, resulting in the formation of a covalent bond between ATG12 and ATG7. ATG3 then acts as an E2 facilitating the binding of ATG12 to ATG5. In the last step, ATG16 is attached forming ATG12-ATG5-ATG16, also known as the ATG16L1 complex (66–68). WIPI2b, a WIPI2 isoform, directly interacts with ATG16L1 and recruits the complex to the phagophore, where ATG16L1 acts similar to E3 enzymes to promote the lipidation of ATG8 family proteins (65).

The ATG8 lipidation machinery contains six ATG8 family proteins, which can be divided into two subfamilies: the microtubule-associated protein light chain 3 (LC3A, LC3B, LC3C) and gamma-aminobutyric acid receptor-associated protein (GABARAP, GABARAPL1, GABARAPL2) (69–71). LC3 is one of the most well-known markers of autophagosomes (72, 73). Analogous to the conjugation of ubiquitin to target proteins, lipidation occurs through the conjugation of phosphatidylethanolamine (PE) to ATG8 proteins such as LC3. ATG4, a cysteine protease, cleaves the C-terminal tail of LC3 exposing a glycine residue. This residue then interacts with the E1-like ATG7 and is conjugated to PE by the E2-like ATG3. To fulfill its function, ATG3 requires stimulation by the ATG16L1 complex acting in an E3-like manner. Lipidation transforms LC3-I (nascent and cytosolic) to LC3-II (conjugated to PE and attached to the autophagosome) (22, 65, 68) (**Figure 1**, panel 3).

ATG4 can also induce deconjugation of ATG8 proteins from PE and must be tightly regulated to prevent autophagosome biogenesis from stalling. This process might be regulated by reactive oxygen species (ROS) generated in mitochondria (74) or by phosphorylation as shown in yeast (75).

Lipids required for ATG8 lipidation and membrane extension are delivered to the phagophore via ATG2 and ATG9, where ATG9 acts as a seed to initiate lipid transfer by ATG2 that acts as a hose funneling lipids from relevant compartments (e.g., the ER) to the phagophore (52–54, 76–78) (**Figure 1**, panel 3). ATG9 may also promote redistribution of the lipids to the inner layer through lipid scramblases (52, 79). ATG8 lipidation facilitates further membrane expansion by recruiting the rest of the autophagic machinery as detailed below or by linking cargo receptors to phagophores during selective autophagy via LC3 interacting regions (LIRs) (40).

After the phagophore has reached its final size, VMP1 promotes the dissociation of contacts between the ER and the phagophore via local changes in Ca^2+^ concentrations until the final scission event is then mediated by the endosomal sorting complexes required for transport (ESCRT) machinery (80–83) (**Figure 1**, panel 3).

#### 1.1.4 Fusion and degradation

For an autophagosome to successfully complete its life cycle, it needs to recruit proteins that facilitate travel from the PAS to the perinuclear region, where autophagosomes fuse with lysosomes. ATG8 family proteins link autophagosomes to kinesins via kinesin adaptors, such as FYVE and coiled-coil domain containing protein 1 (FYCO1) (84). Kinesins, but also dynein motor proteins then mediate travel to the lysosome via microtubules. ATG8 proteins are also responsible for the recruitment of the homotypic fusion and protein sorting (HOPS) complex, a tethering factor required for autophagosome-lysosome fusion (85). The fusion between autophagosomes and lysosomes is driven by the coordinated action of two distinct soluble N-ethylmaleimide-sensitive factor attachment protein receptor (SNARE) complexes (STX17-SNAP29-VAMP8 and YKT6-SNAP29-STX7), tethering factors (e.g., the HOPS complex) and Ras-associated binding proteins (RABs) (e.g., Rab7) (81, 86–89) (**Figure 1**, panel 4). The exact mechanism of fusion between autophagosomes and lysosomes is heavily regulated and has been thoroughly reviewed (81, 86, 90).

Finally, after the fusion of mature autophagosomes with lysosomes, autophagic cargo is degraded by lysosomal hydrolases. This process allows recycling of essential nutrients such as amino acids but also elimination of potentially pathogenic substances (91).

### 1.2 Selective autophagy

During the process of selective autophagy, the autophagic machinery is targeted to specific cargos via selective autophagy receptors independent of the energy sensing pathways mTORC1 and AMPK. Selective autophagy can be classified based on different types of cargo into mitophagy (damaged mitochondria), aggrephagy (protein aggregates), xenophagy (intracellular pathogens), and many more (3).

Cargo selectivity is conferred by selective autophagy receptors. The majority of selective autophagy receptors are soluble proteins referred to as sequestosome-like cargo receptors (SLRs). Among them the most prominent ones are SQSTM1/p62, NDP52, next to BRCA1 gene 1 protein (NBR1), Tax1-binding protein 1 (TAX1BP1) or optineurin (OPTN). Selective autophagy and its receptors have been reviewed expertly elsewhere (3, 40, 92–95). In general, their primary function is to create a direct link between the cargo and the autophagic machinery allowing their selective degradation in the lysosome.

These receptor proteins are characterized by the presence of LIR motifs for the interaction with ATG8 family proteins such as LC3 on the surface of phagophores, and many have ubiquitin-binding domains capable of recognizing ubiquitinated cargo, thus establishing a bridge between the cargo and the autophagic machinery (3, 40, 92, 93). Selective autophagy receptors such as NDP52, TAX1BP1 and p62 are also capable of directly recruiting the ULK1/2 complex through binding to FIP200 (subunit of the ULK1 complex) to initiate *de novo* autophagosome biogenesis at the site of the cargo (40, 42, 43). Moreover, OPTN can directly recruit ATG9A or TANK-binding kinase 1 (TBK1), while NIX can recruit WIPI2, resulting in mitophagy induction (96–98).

A special role during the process of selective autophagy is allocated to TBK1. Initially it was thought that the main role of TBK1 during selective autophagy was facilitating the interaction between ATG8 family protein members and the selective cargo via selective autophagy receptors. We know now that the role of TBK1 in regulating selective autophagy is much broader than previously thought with recent studies implicating TBK1 in the direct recruitment of cargo receptors as well as the early autophagic machinery to intracellular bacteria and mitochondria, which will be described in more detail below (40, 99).

## 2 cGAS-STING signaling

One of the main defense systems against pathogenic bacteria or viruses is the innate immune system. It utilizes pattern recognition receptors (PRRs) to detect and respond to various pathogen- or damage-associated molecular patterns (PAMPs or DAMPs) (100). One such innate immune pathway that was discovered over the last two decades is the cGAS-STING signaling cascade, which recognizes cytosolic double-stranded DNA (dsDNA) and triggers the expression of interferons (IFN) and interferon-stimulated genes (ISGs) (101). ISGs boost the body’s defense against disease by inducing the antiviral state. Cyclic GMP-AMP synthase (cGAS) acts as a PRR for cytosolic dsDNA and generates the second messenger 2´3´-cyclic GMP-AMP (cGAMP). cGAMP binds to stimulator of interferon genes (STING), which further activates interferon regulatory factors 3 and 7 (IRF3, IRF7) that drive type I IFN expression or nuclear factor kappa-light-chain-enhancer of activated B-cell (NF-κB) that induces the expression of proinflammatory genes (101–104).

Chronic or heightened activation of cGAS-STING signaling has been associated with autoimmune diseases such as Aicardi-Goutières syndrome and other pathologies. Additionally, it is also a prominent target for cancer research due to its interplay with many chemotherapeutic treatments, radiation therapy and immune checkpoint inhibitors, which could lead to abscopal responses and improve patient outcome (105, 106).

Recent reports have linked the cGAS-STING pathway with autophagy, revealing multiple regulatory mechanisms and interactions that highlight the potential of exploiting these pathways for cancer therapy.

### 2.1 The mechanism of cGAS-STING signaling

**Figure 2:**
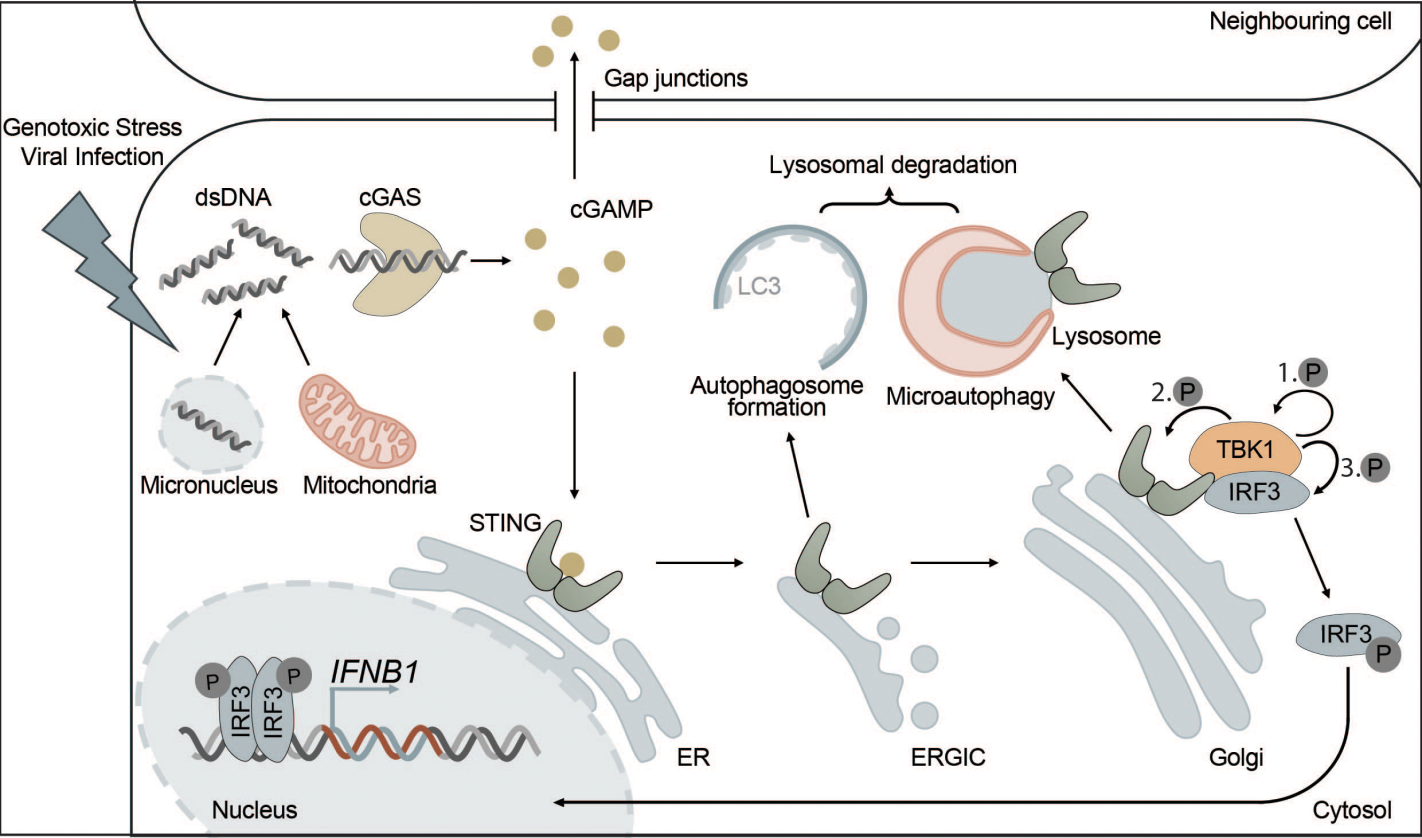
The cGAS-STING pathway is directly connected to autophagy. cGAS-STING signaling is an innate immune pathway, responsible for the production of type I IFNs. Extracellular stressors such as genotoxic stress or viral infection as well as genomic instability can lead to the appearance of dsDNA in the cytosol. cGAS is activated upon binding dsDNA, which leads to the production of the secondary messenger cGAMP. cGAMP then activates STING, located at the ER, inducing its oligomerization and translocation to the Golgi via the ERGIC. Alternatively, cGAMP can also trigger the activation of STING in neighbouring cells via intercellular transport mediated by gap junctions. At the Golgi, STING induces the phosphorylation of TBK1 and ultimately IRF3 leading to its translocation to the nucleus where phosphorylated IRF3 drives the transcription of type I IFNs. Each distinct step of the cGAS-STING pathway is interconnected with autophagy. For example, STING, upon activation, leads to the formation of autophagosomes at the ERGIC in a WIPI2-dependent manner. This acts as a negative feedback loop by degrading dsDNA and other members of the pathway, such as cGAS or IRF3. Additionally, microautophagy is responsible for the final termination of STING signaling in an ESCRT-dependent manner.

Under physiological conditions, DNA is exclusively found in the nucleus and in mitochondria. However, certain types of cellular stress can lead to the accumulation of DNA in the cytosol, where it is then recognized by cGAS. Cytosolic DNA may originate from DNA viruses, intracellular bacteria or RNA viruses causing the release of mitochondrial DNA (mtDNA) (101, 107–111). In addition, cGAS can recognize not only foreign DNA but also endogenous DNA that is released into the cytosol from mitochondria or micronuclei resulting from mitotic defects (**Figure 2**). Extracellular DNA originating from dead cells can also be taken up by cells and stimulate the cGAS-STING pathway (111–116). cGAS is also found in the nucleus but is kept inactive due to nucleosome binding and chromatin tethering (117). However, nuclear cGAS can be activated by human immunodeficiency virus (HIV) in dendritic cells and macrophages (118).

Structurally, cGAS can bind double-stranded DNA as well as single-stranded DNA with secondary structures and has even been shown to bind RNA-DNA hybrids (119–122). Even though both small and large DNA fragments can be bound, cGAS activation requires a certain minimal fragment size and has been shown to improve with increasing DNA length (120–124). A very recent publication proposes that mechanical flexibility of DNA, which is increased in the case of DNA damage and higher AT content, promotes the binding and activation of cGAS (125).

Upon DNA binding, cGAS undergoes a conformational change, leading to activation and dimerization with two DNA molecules sandwiched in between (124, 126–128). Those dimers can undergo liquid-liquid phase separation in a DNA length-dependent manner, forming condensates that act as “minireactors” and concentrate cGAS as well as substrates to increase the catalytic activity of cGAS. This could also explain why longer DNA fragments activate cGAS more efficiently (129).

Once activated, cGAS uses GTP and ATP as substrates to produce the secondary messenger cGAMP, which can then be recognized by the ER transmembrane protein STING (130–132). Additionally, cGAMP can diffuse into neighboring cells via gap junctions or be released into the local microenvironment to activate STING signaling in surrounding cells (133–137).

STING resides at the ER membrane as a dimer, where two C-terminal ligand-binding domains form a binding pocket for cGAMP. Binding of cGAMP induces a conformational change that facilitates oligomerization of STING dimers and STING activation (138–140).

Active STING is translocated from the ER to the ER-Golgi intermediate compartment (ERGIC) and Golgi via COPII vesicles, which requires secretion-associated and Ras-related GTPase 1A (SAR1), SEC24 homolog C (SEC24C) and ADP-ribosylation factor (ARF) family members (141).

At the Golgi and in post-Golgi compartments, TBK1 is recruited and then activated by STING (142). Activated TBK1 phosphorylates the C-terminal tail (CTT) of STING at several residues including Ser366, which is part of a highly conserved motif (pLXIS) (143, 144). This motif recruits IRF3, which is then phosphorylated by TBK1, inducing its dimerization. Phosphorylated IRF3 dimers can translocate into the nucleus where they act as transcription factors for type I interferons and other immune-related proteins (143–145) (**Figure 2**). After activation of IRF3, cGAMP-bound STING oligomers are transported through the trans-Golgi and sorted into RAB7-positive late endosomes that travel to lysosomes for degradation via multivesicular bodies (141).

In addition to its function as an activator of type I IFN synthesis and autophagy, STING can also activate the NF-κB pathway in a TBK1-IKKϵ-dependent or TBK1-independent manner that involves DNA damage response factors ataxia telangiectasia mutated (ATM) and poly(ADP-ribose) polymerase 1 (PARP1) (146–148).

## 3 Interactions of autophagy with the cGAS-STING pathway

### 3.1 cGAS

**Figure 3:**
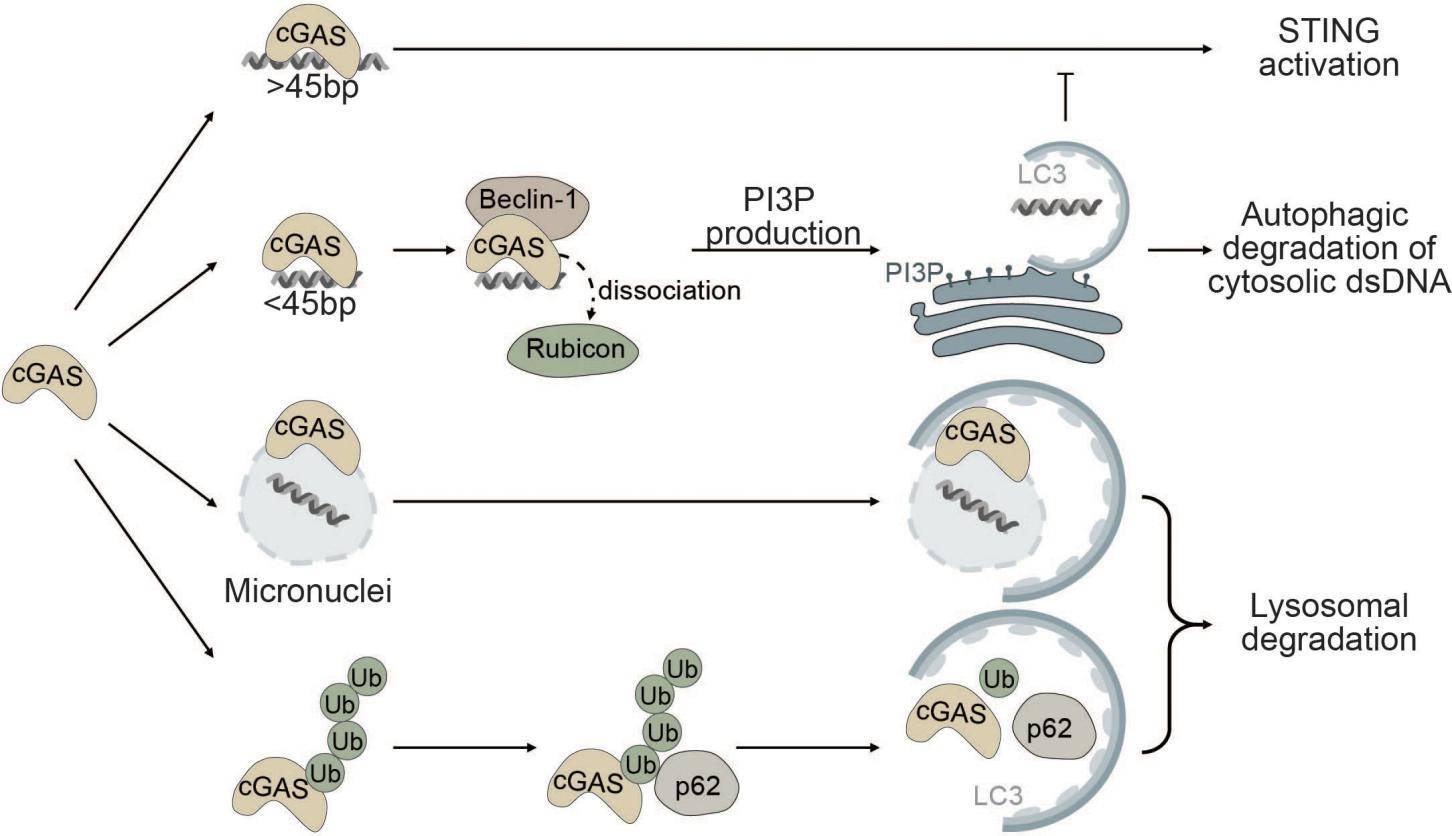
Autophagy as a negative regulator of cGAS. cGAS is a cytosolic sensor for long cytosolic dsDNA fragments. Binding to long dsDNA fragments leads to productive cGAS-STING signaling culminating in the production of type I IFNs. Conversely, binding of small cytosolic dsDNA (<45bp) promotes binding of cGAS to Beclin-1, thereby reducing the interaction of Beclin-1 with Rubicon, a negative regulator of autophagy. This gives rise to the production of PI3P and the formation of autophagosomes allowing degradation of cytosolic dsDNA and negatively impacting cGAS activation. cGAS can also act as a selective autophagy receptor for micronuclei causing their ultimate degradation and thereby prohibiting them from releasing DNA into the cytosol. Finally, K48-linked ubiquitination of cGAS promotes selective p62-dependent autophagic degradation of cGAS, limiting the available amount of activatable cGAS.

The interplay between autophagy and cGAS occurs through (i) cGAS-Beclin-1 interaction that promotes autophagic degradation of cytosolic dsDNA, (ii) cGAS-LC3 interaction that promotes micronucleophagy, and (iii) degradation of cGAS through p62-mediated selective autophagy (**Figure 3**).

Robust activation of the enzymatic function of cGAS is dependent on the length of dsDNA fragments and longer fragments (>45bp) promote robust activation of cGAS (123, 149). Conversely, small cytosolic dsDNA fragments decrease cGAS-STING signaling by competing with long dsDNA fragments for cGAS binding. Binding of small dsDNA fragments to cGAS promotes the binding of cGAS to Beclin-1. By binding to Beclin-1, cGAS outcompetes run domain Beclin-1 interacting and cysteine-rich containing protein (Rubicon) (150). Rubicon negatively regulates maturation and fusion of the autophagosome with the lysosome and endocytic trafficking by suppressing VPS34 kinase activity (151–153). Binding of cGAS to Beclin-1 leads to the release of Rubicon, thereby enabling autophagic degradation of cytosolic dsDNA and resulting in decreased cGAS-STING signaling (**Figure 3**). Moreover, Beclin-1 also suppresses the NTase activity of cGAS to decrease the production of the secondary messenger cGAMP and type I IFN production (150, 154).

Furthermore, cGAS itself has been proposed as a potential selective autophagy receptor for autophagic degradation of micronuclei also known as micronucleophagy (155). Overexpression of cGAS significantly reduces the number of micronuclei in an autophagy-dependent manner independent of its role in cGAS-STING signaling. cGAS was shown to directly interact with the ATG8 family protein LC3 and colocalize at micronuclei, thereby facilitating the recruitment of the autophagic machinery to enable lysosomal degradation of micronuclei (**Figure 3**). Moreover, cGAS-driven micronucleophagy also dampens cGAMP production and altogether exerts a negative effect on cGAS-STING signaling and innate immunity (155).

In another report, it was shown that K48-linked ubiquitinated cGAS is directly sequestered into the lysosome for degradation by interacting with the selective autophagy receptor p62 (**Figure 3**). Upon viral infection, the ISG tripartite motif 14 (TRIM14) directly binds to the C-terminus of cGAS and recruits ubiquitin-specific peptidase 14 (USP14) to prevent K48-linked ubiquitination and degradation of cGAS. Consequently, cGAS evades p62-mediated autophagic degradation and can stimulate type I IFN signaling after viral infection (156).

### 3.2 STING

**Figure 4:**
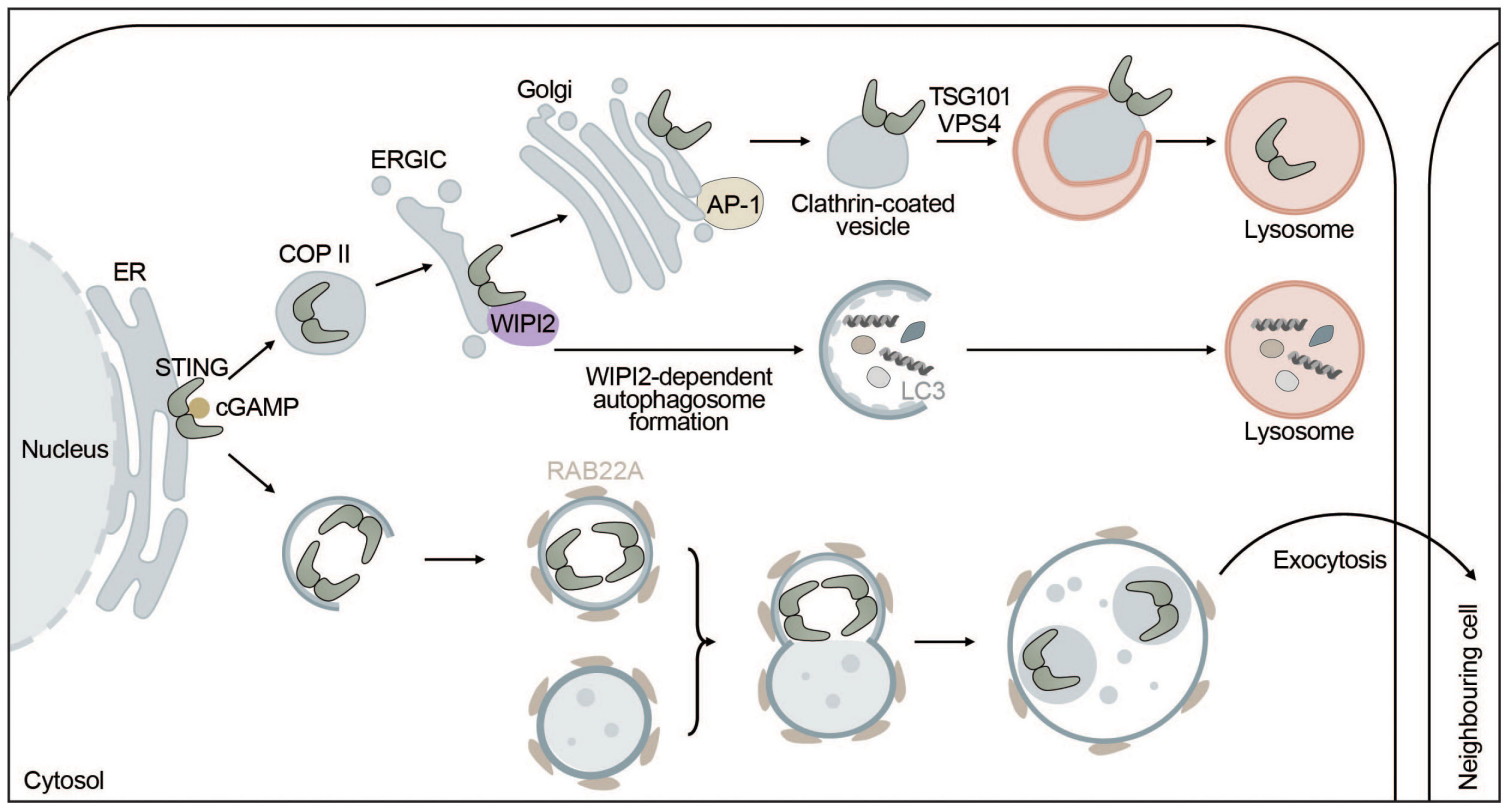
STING signaling is linked to autophagy. STING directly induces the formation of autophagosomes at the ERGIC in a WIPI2-dependent manner utilizing the ERGIC as a membrane source. This process is independent of “canonical” upstream autophagic machinery such as ULK1 or VPS34. However, STING itself is not degraded this way but rather by microautophagy where it is packaged into clathrin-coated vesicles from the Golgi by AP-1. These vesicles are directly taken up by lysosomes thereby terminating STING signaling. Alternatively, upon activation by cGAMP, STING can be transported to neighbouring cells inside non-canonical RAB22A-dependent autophagosomes that fuse with endosomes. The emerging new vesicle can inactivate Rab7, thereby escaping lysosomal degradation and allowing STING pathway activation in surrounding tissues.

STING and autophagy are connected on four levels: (i) STING facilitates non-canonical induction of autophagy, (ii) microautophagy and selective macroautophagy can induce STING degradation, (iii) ULK1 negatively regulates STING-IRF3 signaling by phosphorylating STING, and (iv) non-canonical RAB22-mediated autophagy can promote intercellular spreading of activated STING (**Figure 4**).

Autophagy induction has been proposed to be the primordial function of cGAS-STING signaling before interferon induction evolved. STING can induce autophagy independent of TBK1 and IRF3 via an LR motif at the C-terminus of STING (141, 157). This motif can also be found in other organisms such as *Nematostella vectensis* that are lacking the C-terminal activation domain required for interferon production. Upon cGAMP binding, STING moves to the ERGIC in a COPII- and ARF GTPase-dependent manner. The ERGIC can then serve as a membrane source for LC3 lipidation, which is dependent on the adaptor protein WIPI2 and ATG5 but does not require ULK1 or VPS34, indicating that this non-canonical STING-dependent induction of autophagy can “skip” early steps of the canonical autophagy mechanism (141). STING directly recruits WIPI2 to STING-positive vesicles and competes with PI3P (canonical bulk autophagy) for binding to WIPI2, adding another layer of complexity to the feedback mechanism between autophagy and cGAS-STING signaling (158) (**Figure 4**). STING-induced non-canonical autophagy was shown to inhibit herpes simplex virus (HSV-1) (159).

Myristic acid has recently emerged as a regulator of STING degradation via N-myristoylation of ARF1, which is involved in directing the membrane trafficking and autophagic degradation of STING, thereby limiting cGAS-STING-induced interferon response. Upon viral infection the production of myristic acid is reduced due to a disruption in the cellular lipid metabolism, which alleviates autophagy-dependent STING degradation (160).

Another recent report postulated that STING-induced LC3 lipidation happens independent of WIPI2 at perinuclear single membrane vesicles, rather than double-membrane autophagosomes, and requires both the V-ATPase as well as the WD40 domain of ATG16L1 required for interaction with V-ATPase (161, 162). Autophagy-independent LC3 lipidation occurs during influenza A virus infection and mouse models lacking the WD40 domain of ATG16L1 show a reduction in major histocompatibility complex class II (MHC-II)-dependent antigen presentation in dendritic cells (162). The V-ATPase-ATG16L1 axis was also shown to initiate intracellular clearance of bacteria (163). In line with this, STING was recently shown to have proton channel activity that is essential for both non-canonical LC3 lipidation and inflammasome activation. This would allow for mechanistic decoupling of IFN induction via phosphorylated STING and proton channel-dependent LC3 lipidation and inflammasome activation (164). In conclusion, STING can induce non-canonical autophagy in a WIPI2-dependent manner at the ERGIC and autophagy-independent LC3 lipidation at perinuclear single membrane vesicles facilitating defense against viruses and bacteria.

While STING can induce autophagy, autophagy can degrade STING through multiple mechanisms including microautophagy and selective macroautophagy. In microautophagy, cytoplasmic cargo is directly engulfed by the lysosome. STING is transported by clathrin-coated vesicles and recycling endosomes (141) and directly encapsulated in lysosomal associated membrane protein 1 (LAMP-1) positive compartments through the ESCRT components tumor susceptibility gene 101 (TSG101) and vacuolar sorting-associated protein 4 (VPS4) (165). STING packaging from the trans-Golgi into clathrin-coated vesicles is mediated by the cargo adaptor complex 1 (AP-1), which also controls the termination of STING-dependent immune activation by an as of yet unknown mechanism (166) (**Figure 4**). In addition, basal STING levels seem to be kept under control by the ER-associated degradation (ERAD) system. The ERAD protein complex SEL1L-HRD1 directly interacts with and mediates targeted degradation of basal inactive STING thereby limiting the activatable STING pool and preventing overactivation of the immune response (167). Alternatively, STING may be degraded through selective macroautophagy, whereby TBK1-mediated phosphorylation of p62 at S403 increases its affinity for K63-ubiquitinated STING (168) and ubiquitously expressed transcript (UXT) may facilitate the interaction between p62 and STING (169). Taken together, microautophagy and p62-mediated selective autophagy regulate STING degradation and thereby keep the interferon response in check.

Autophagy has also been shown to have a more direct function in negatively regulating STING-dependent signaling. ULK1 phosphorylates STING at S366, thereby inhibiting IRF3- but not NF-κB-dependent interferon production upon stimulation with dsDNA (170).

A non-canonical autophagy pathway that relies on RAB22A was shown to facilitate the spreading of activated STING to neighboring cells. RAB22A activates phosphatidylinositol 4-phosphate 3-kinase C2 domain-containing subunit alpha (PI3K2A) to produce phosphatidylinositol 4-phosphate (PI4P) that recruits ATG12-ATG5-ATG16L1. The ATG16L1 complex then induces the formation of ER-derived non-canonical autophagosomes containing activated STING. RAB22A-induced autophagosomes fuse with early endosomes, while inhibiting RAB7-mediated fusion with lysosomes. Secretion of the RAB22A-induced extracellular vesicles enables intercellular transfer of activated STING and the spreading of interferon induction to neighboring cells similarly to cGAMP (171) (**Figure 4**).

In summary, STING can induce non-canonical formation of autophagosomes via WIPI2 using the ERGIC as a source for autophagosome lipidation and promotes autophagy-independent LC3 lipidation. However, none of these pathways seem to mediate the degradation of STING. Instead, STING is degraded by direct microautophagic uptake by lysosomes after being packaged into clathrin-coated vesicles, which is mediated by AP-1. In addition to microautophagic degradation, p62-dependent degradation of STING might also contribute to this phenomenon. Lastly, STING can be taken up by non-canonical RAB22A-induced autophagosomes that do not fuse with lysosomes but are secreted to surrounding cells, spreading active STING. Overall, STING positively regulates and induces different types of autophagy, while autophagy negatively impacts STING signaling (**Figure 4**).

### 3.3 TBK1

**Figure 5:**
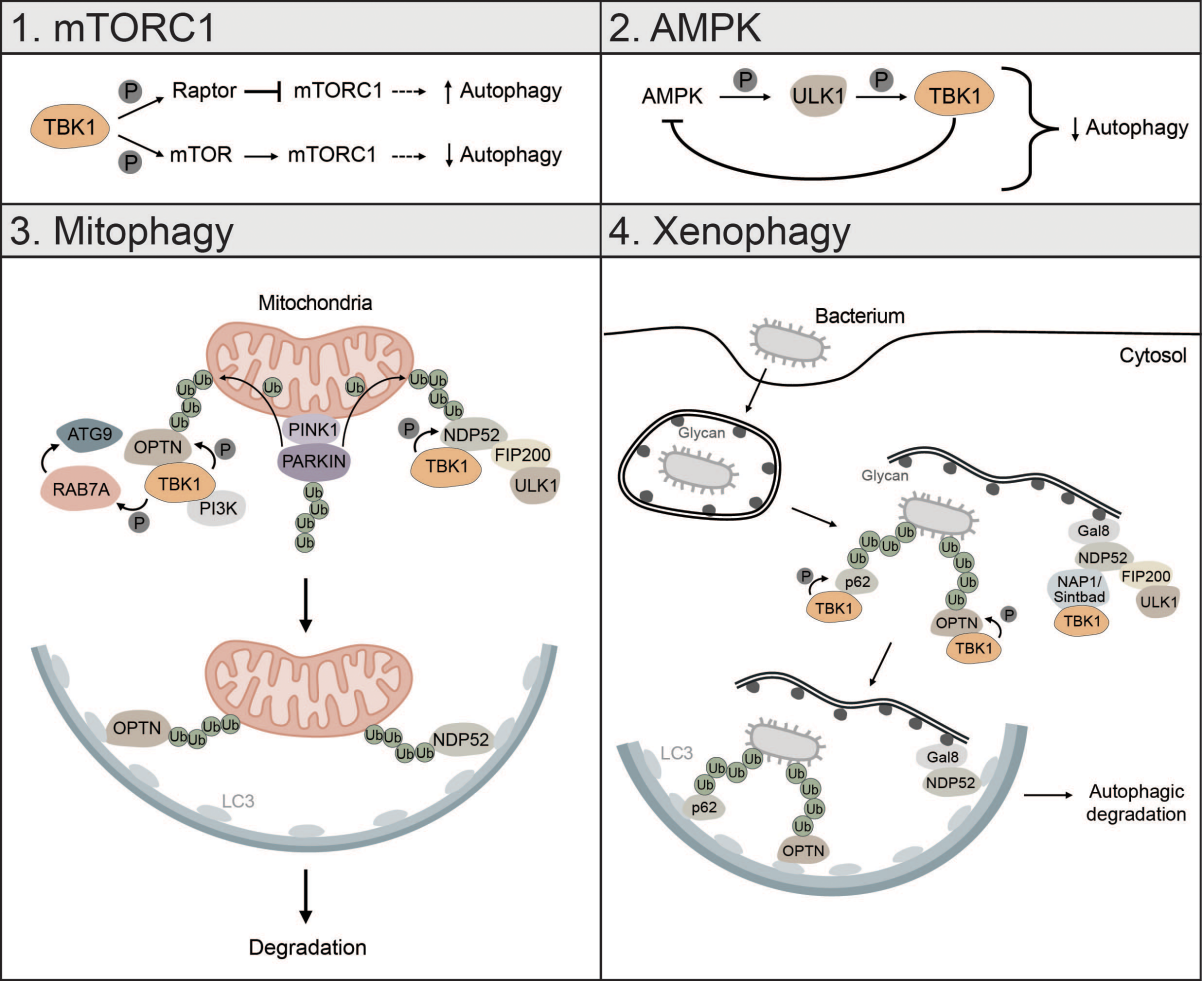
TBK1 regulates different types of autophagy. 1) mTORC1: TBK1 can positively and negatively affect autophagic flux via direct or indirect regulation of mTORC1. Phosphorylation of RAPTOR by TBK1 decreases the activity of mTORC1 and promotes autophagy. In addition, TBK1 can directly phosphorylate mTOR, which increases mTORC1 activity and decreases autophagic flux. 2) AMPK: AMPK can enhance TBK1 activity via ULK1-mediated phosphorylation of TBK1. High levels of active TBK1, however, negatively regulate AMPK, thus creating a negative feedback loop that can negatively affect autophagic flux. 3) Mitophagy: During PINK1-PARKIN-mediated mitophagy, OPTN and NDP52 redundantly activate TBK1, which in turn phosphorylates OPTN and NDP52 to ensure their retention at damaged mitochondria and facilitate the recruitment of ATG8 family proteins. In addition, TBK1-mediated phosphorylation enables spatial proximity to the autophagic machinery by facilitating the binding of NDP52 to the FIP200/ULK1 complex. During OPTN-induced autophagy, TBK1 can take over the functions of the ULK1 complex and directly interact with the PI3K complex to drive mitophagy. Moreover, by phosphorylating RAB7A, TBK1 enables recruitment of ATG9-positive vesicles to damaged mitochondria. 4) Xenophagy: Gal8 recognizes exposed glycans and recruits NDP52. Subsequently, TBK1 is recruited via the two adapter proteins NAP1 and SINTBAD leading to the formation of a NDP52-ULK1-TBK1 super complex, which is essential for phagophore formation. In addition, TBK1 directly phosphorylates the selective autophagy receptors p62 and OPTN, which are crucial for autophagic clearance of ubiquitin-coated bacteria.

The kinase TBK1 is known to fulfill a broad range of functions in our cells ranging from IFN signaling, innate immunity and inflammation to autophagy. It is likely that distinct functions of TBK1 in different cellular pathways are due to its cellular localization and association with different adaptor proteins (99, 172). Moreover, TBK1 may simultaneously regulate autophagy and IFN signaling by controlling starvation-induced autophagy and different forms of selective autophagy (e.g., mitophagy or xenophagy). TBK1 may regulate the clearance of dsDNA in the cytosol and thereby modulate cGAS-STING activation and IFN production. TBK1 can regulate autophagy by: (i) activating or inhibiting mTORC1, (ii) inhibiting AMPK, (iii) promoting mitophagy, and (iv) promoting xenophagy (**Figure 5**).

During starvation-induced autophagy, TBK1 can positively and negatively regulate autophagic flux by inhibiting or activating mTORC1 respectively (173, 174). TBK1 can phosphorylate and activate regulatory-associated protein of mTOR (RAPTOR), which decreases activity of mTORC1 and promotes autophagy (173). Besides inhibition of mTORC1, TBK1 can directly activate mTORC1 by phosphorylation of mTOR on S2159 to control innate immunity (174) (**Figure 5**). Whether TBK1 activates or inhibits mTORC1 seems to be cell-context dependent. TBK1 can also initiate autophagy under starvation conditions by phosphorylating and activating the autophagosome-lysosomal fusion protein syntaxin 17 (STX17). Phosphorylated STX17 translocates from the Golgi to the mammalian PAS and controls the formation and assembly of the ULK1 complex, thereby promoting initiation of the autophagic process (175).

The energy sensor AMPK is also regulated by TBK1. Mice with high fat diet have high protein levels of TBK1, which negatively regulates AMPK activity. At the same time, active AMPK indirectly enhances TBK1 activity by promoting ULK1-mediated phosphorylation of TBK1. This creates a negative feedback loop whereby AMPK activates TBK1, which downregulates AMPK, possibly explaining diminished obesity in TBK1 KO mice (176). Since AMPK is known to promote autophagy via direct activation of ULK1 (24), this negative feedback loop between TBK1 and AMPK can negatively affect autophagic flux (**Figure 5**).

TBK1 is also involved in the regulation of mitophagy, especially in the early steps, where it is instrumental for initiating autophagosome biogenesis (43, 97, 177, 178). Mitophagy is activated by PTEN-induced kinase I (PINK1), which upon mitochondrial depolarization accumulates at the outer mitochondrial membrane (OMM) followed by the recruitment of PARKIN and ubiquitination of mitochondrial proteins (40, 179). During PINK1-PARKIN-induced mitophagy, the selective autophagy receptors OPTN and NDP52 redundantly activate TBK1, which in turn phosphorylates the mitophagy receptors OPTN, NDP52 and p62 to ensure their retention at damaged mitochondria and to facilitate the recruitment of ATG8 family proteins (180, 181). TBK1-mediated phosphorylation of NDP52 facilitates direct interaction with the FIP200/ULK1 complex thus enabling spatial proximity to ubiquitinated cargo to drive autophagosome biogenesis (43). OPTN-induced mitophagy is not necessarily dependent on the binding to the FIP200/ULK1 complex as shown for NDP52. In fact, TBK1 takes over the functions of the ULK1 complex and directly interacts with the PI3K complex I to initiate mitophagy (97). TBK1-mediated phosphorylation of OPTN supports mitophagy by stabilizing the binding of OPTN to ubiquitinated cargo (181). Reciprocally, OPTN-mediated TBK1 relocalization to mitochondria is crucial for proper TBK1 activation (182, 183). TBK1 also phosphorylates RAB7A, which promotes the recruitment of ATG9-positive vesicles to damaged mitochondria enabling efficient mitophagy (184) (**Figure 5**). Recently, it was shown that mitochondria can be cleared upon PINK1-PARKIN mediated mitophagy in the absence of the mATG8-conjugation machinery and independent of lysosomal degradation via a process called autophagic secretion of mitochondria (ASM). ASM promotes cGAS-STING signaling in recipient cells. The exact mechanism of how mtDNA-containing extracellular vesicles get in contact with cytosolic cGAS of recipient cells remains to be elucidated (185).

Xenophagy is another form of selective autophagy responsible for elimination of pathogens or clearance of invading bacteria which, like mitophagy, relies on TBK1 (3). During early xenophagy, Galectin-8 (GAL8) recognizes exposed glycans on ruptured vacuoles and subsequently recruits the selective autophagy receptor NDP52. Binding of NDP52 to the two adaptor proteins NAP1 and SINTBAD enables recruitment of TBK1 and the formation of an NDP52-ULK1-TBK1 super complex, which is essential for WIPI2-positive phagophore formation and for the recruitment of LC3 (42) (**Figure 5**). In addition, the ubiquitin ligase RNF213 directly ubiquitylates lipopolysaccharides (LPS) upon vacuole rupture, followed by the recruitment of linear (M1) Ub assembly complex (LUBAC), which is responsible for the assembly of M1 ubiquitin chains. The recruitment of the selective autophagy receptor OPTN and the immune adaptor NF-κB essential modulator (NEMO) is dependent on LUBAC function, while the recruitment of the selective autophagy receptors p62 and NDP52 requires only RNF213 (186). TBK1 directly phosphorylates S403 of p62, which is required for the autophagic function of p62 in driving autophagosome maturation (187). OPTN is also directly phosphorylated by TBK1, thereby enhancing LC3 binding and autophagic clearance of ubiquitin-coated bacteria (188) (**Figure 5**).

**Figure 6:**
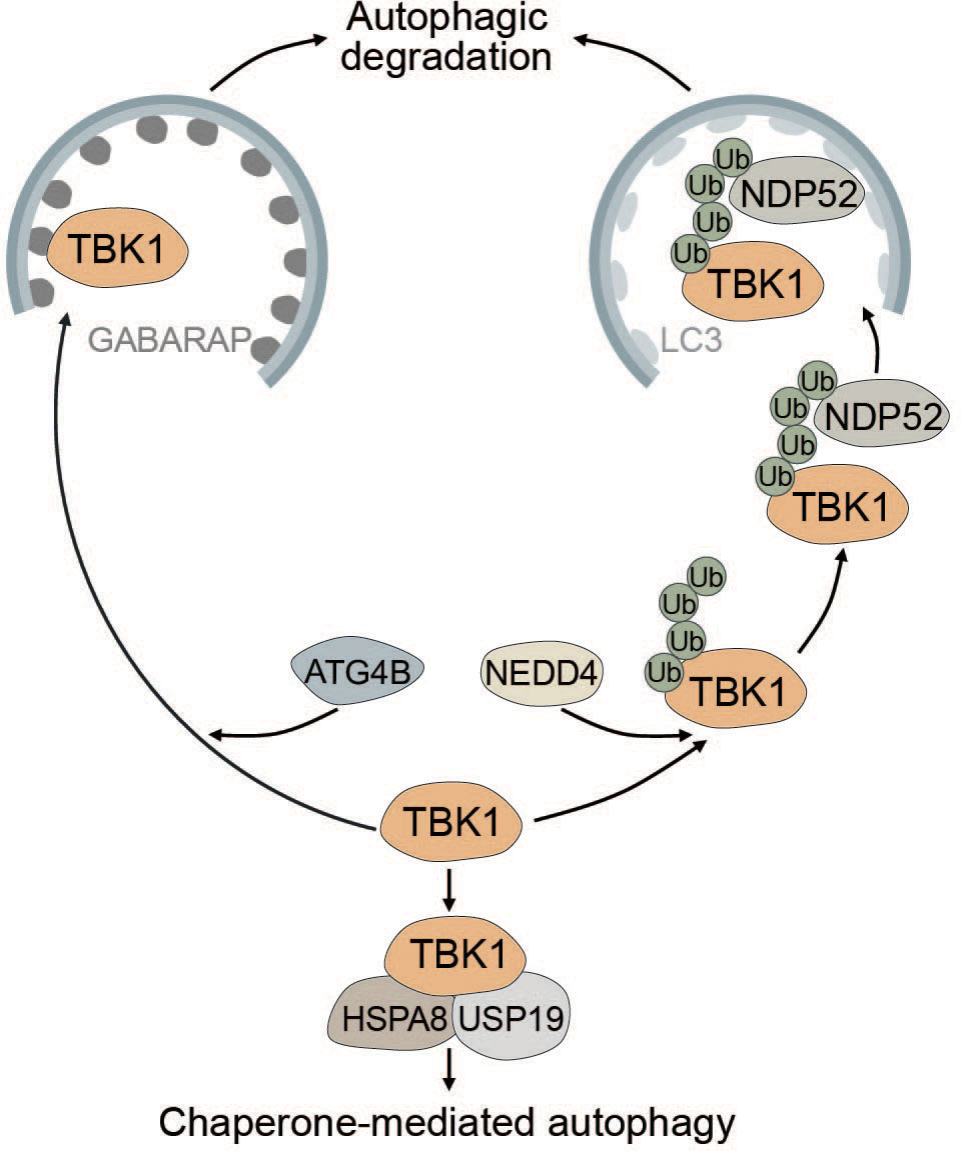
Multiple modes of autophagy negatively regulate TBK1. ATG4B directly associates with TBK1 and acts as an adapter mediating the direct LIR-dependent interaction between the ATG8 family protein GABARAP and TBK1 leading to TBK1 degradation via the autophagosome. The E3 ubiquitin ligase NEDD4 is responsible for the deposition of K27-linked polyubiquitin chains on TBK1, which can be recognized by NDP52 causing selective degradation of TBK1. Conversely, TBK1 can also be degraded by chaperone-mediated autophagy via TBK1 CMA motif that is recognized by the CMA adapter proteins HSPA8 and USP19.

TBK1 is known to be tightly regulated by multiple post-translational modifications to prevent sustained and prolonged immune activation (99, 189–191). The E3 ubiquitin ligase neural precursor cell-expressed developmentally downregulated gene 4 (NEDD4) controls the stability of TBK1 through K27-linked polyubiquitination. The selective autophagy receptor NDP52 binds polyubiquitinated TBK1 and elicits autophagic degradation, resulting in decreased type I IFN signaling (189) (**Figure 6**). Lysosomal degradation of TBK1 is also promoted by ubiquitin specific peptidase 19 (USP19), which interacts with the TBK1-HSPA8 complex via the chaperone-mediated autophagy (CMA) motif of TBK1 (190) (**Figure 6**). Another way in which autophagy negatively regulates TBK1 stability is through the autophagy protein ATG4B, which serves as an adaptor for the recruitment of TBK1 to gamma-aminobutyric acid receptor-associated protein (GABARAP) via its LIR motif. Subsequently, TBK1 is sequestered into the lysosome and degraded (**Figure 6**). Pharmacological inhibition of ATG4B-dependent autophagic degradation of TBK1 was shown to increase the overall antiviral response (192). Taken together, TBK1 stability is regulated by autophagy to prevent sustained type I IFN production and prolonged immune reaction.

### 3.4 IRF3

**Figure 7:**
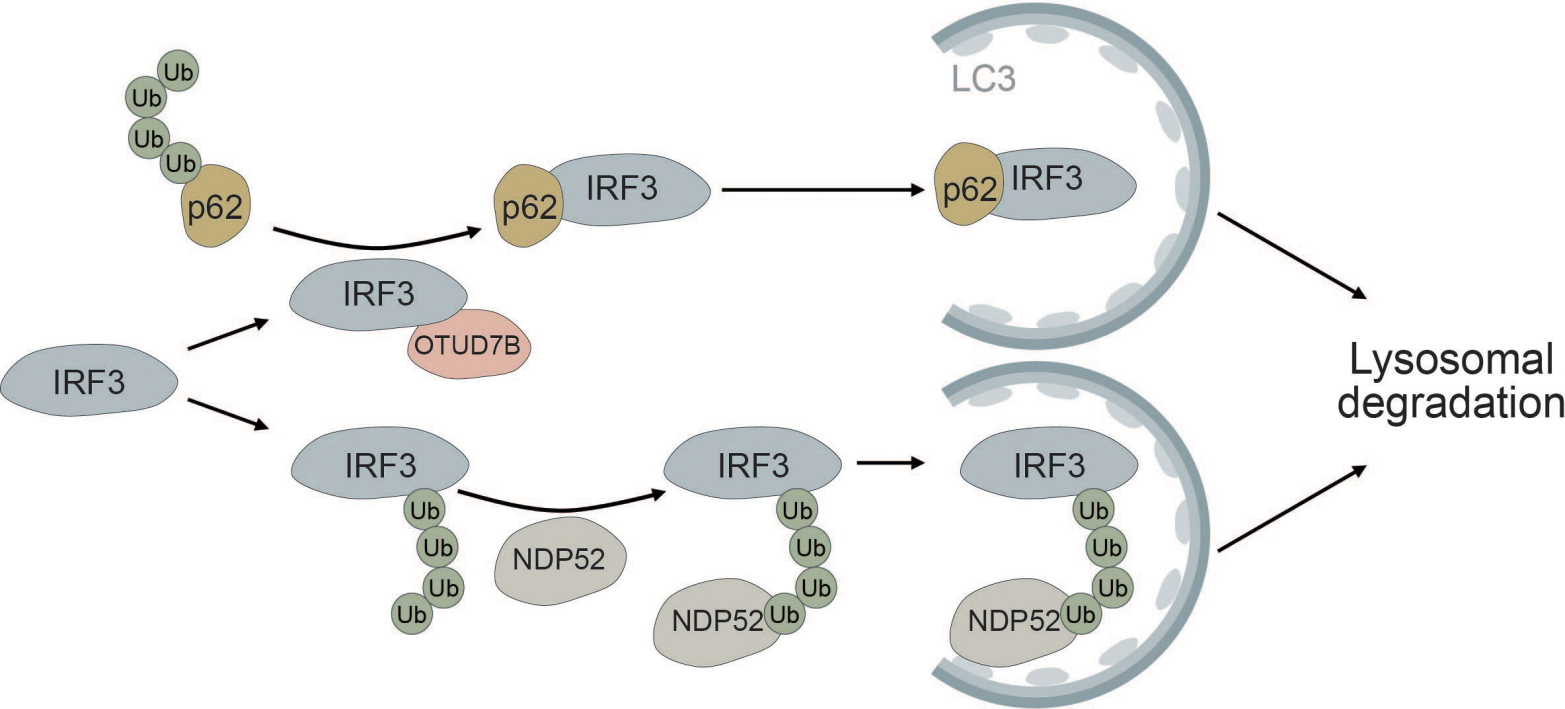
IRF3 is selectively degraded via the autophagic cargo receptors p62 and NDP52. The deubiquitinase OTUD7B removes ubiquitin chains from p62, thereby allowing it to more efficiently oligomerize. This allows for tighter association between p62 and IRF3, causing selective autophagic degradation of IRF3. Autophagic degradation of IRF3 is further promoted by the selective cargo receptor NDP52, which recognizes K27-linked polyubiquitin chains on IRF3, thereby inducing its autophagic degradation.

Autophagic degradation of IRF3 is another mechanism of keeping immune activation in check. NDP52 mediates the selective degradation of IRF3 upon viral infection (**Figure 7**). Under homeostatic conditions, the deubiquitinase PSMD14 cleaves K27-linked polyubiquitin chains at K313 of IRF3, thereby preventing autophagic recognition and degradation (193). Furthermore, OUT deubiquitinase 7B (OTUD7B) promotes autophagic degradation of IRF3 upon viral infection by enhancing the association between IRF3 and p62, thereby negatively regulating the immune response. OTUD7B directly interacts with IRF3 and activates p62 by removing K63 polyubiquitin chains leading to enhanced p62 oligomerization. This allows more efficient degradation of IRF3 in a p62-dependent manner (194) (**Figure 7**). The histone deacetylase HDAC10 can bind to IRF3 in a deacetylase-independent manner and inhibit its TBK1-dependent phosphorylation at S396. Viral infection induces targeted autophagic degradation of HDAC10 resulting in increased phosphorylation of IRF3 and enhanced interferon response (195).

## 4 Autophagy and cGAS-STING signaling in cancer

One of the major functions of the immune system is the prevention of malignant transformation, cancer progression as well as metastasis (196, 197). Modern cancer therapies utilize the immune system to target and destroy cancer cells and to provide lasting recovery. One of the main pathways involved in response to radiotherapy and chemotherapies is cGAS-STING signaling. In recent years, many therapies specifically targeting the immune signaling pathway have been tested (198).

Since autophagy is one of the main players in the degradation of damaged organelles and protein aggregates to maintain cellular homeostasis and is also tightly connected to other essential cellular signaling pathways like nutrient sensing or cell death, deregulation of this pathway can have severe consequences in cancer development and progression (19, 199).

Both cGAS-STING signaling and autophagy were shown to form multiple signaling complexes with different cellular effectors that may exert pro- or anti-tumorigenic functions at different stages of the disease (1, 200). We will provide an overview of the involvement of cGAS-STING signaling and autophagy in cancer progression and treatment strategies individually, as well as their interactions in the context of cancer and how this might affect potential therapeutic strategies.

### 4.1 cGAS-STING and cancer

Genomic instability resulting from defects in DNA damage response, chromosomal instability or defects in chromosome segregation can lead to the formation of micronuclei and generation of aberrant cytosolic dsDNA fragments (113, 201, 202). Micronuclei are small nuclei-like structures containing chromatin encapsulated by the nuclear envelope, which arise from chromosomal instability (203). Micronuclei can rupture and release genomic dsDNA into the cytosol, which is directly engaged by cGAS (113, 204). In addition to genomic dsDNA, dsDNA released from mitochondria can also directly activate the cGAS-STING pathway (205). Another way to activate cGAS is through dying tumor cells, which release DNA into their surroundings. This DNA can be taken up by dendritic cells and activate the cGAS-STING pathway (206).

One of the major concepts in cancer biology is the cancer-immunity cycle, a cyclic self-propagating process that leads to the initiation and amplification of T-cell responses against cancer cells (197). Functional STING signaling affects multiple stages of the cancer-immunity cycle and can enhance tumor-specific T-cell responses (197, 198, 207). Type I IFNs produced by activation of the cGAS-STING pathway instigate the differentiation and maturation of antigen-presenting cells (APCs) and the expression of MHC molecules, thereby facilitating enhanced tumor antigen presentation (208–211). Consequently, cGAS-STING signaling is important for priming, activation and infiltration of tumor-specific cytotoxic T cells (212–214). Active cGAS-STING signaling has also been linked with the production of certain chemokines that recruit T cells to the tumor site such as CXCL10, as well as induction of T-cell stemness (197, 215). Generally, the induction of the type I IFN response targets different immune cells from antigen-presenting dendritic cells, T cells to natural killer (NK) cells (200). In addition to the production of type I IFNs, STING activation can induce the NF-κB pathway and the expression of various proinflammatory cytokines such as interleukin-6 (IL-6) and tumor necrosis factor-a (TNF-α) (200). Apart from its intrinsic function in tumor and immune cells, cGAS-STING can signal intercellularly by cGAMP spreading to neighboring cells via gap junctions (134, 216, 217). This process is an active field of study and has recently been reviewed (217). Conventional cancer therapies including radio- and chemotherapy can induce cGAS-STING and anti-tumor immunity alone or in combination with STING agonists and immune checkpoint blockade (198, 218, 219).

In contrast to its anti-tumorigenic function, cGAS-STING signaling has also been implicated in oncogenesis (200, 220–222). The major culprits seem to be chronic type I IFN production and inflammation, which promote cancer cell invasion, metastasis and cancer cell stemness (200, 223–225). Recently, active STING signaling has been linked to the survival of chromosomally unstable cancers in an IL-6 dependent manner involving both NF-κB and signal transducer and activator of transcription 3 (STAT3) activation (226). Additionally, chromosomal instability can promote metastasis via STING-dependent NF-κB activation (227).

Overall, the relationship between pro- and antitumorigenic functions of the cGAS-STING pathway are complex and highly dependent on the genetic makeup of the tumor as well as the tumor microenvironment. Recent therapeutic approaches have mostly focused on the induction of the pathway with some promising results that seem to outweigh the negative effects. However, preventing chronic induction of type I interferons by using short, focused bursts of interferon induction might prove valuable in the future in preventing possible side effects.

### 4.2 Autophagy and cancer

Similar to cGAS-STING signaling, the function of autophagy in cancer development and therapy is complex. The effect of autophagy on cancer is strongly dependent on the stage of the disease. Initially, autophagy is involved in the control and prevention of malignant transformation. Since autophagy mostly acts in a cytoprotective fashion, this also applies to cells that have already turned to malignancy, providing them with multiple tools to avoid detection and destruction (1, 19, 228).

#### 4.2.1 Tumor suppression

In early stages of tumorigenesis autophagy may act as a tumor suppressor by exhibiting various cytoprotective effects such as degradation of damaged organelles or proteins and maintenance of cellular homeostasis. Indeed, several reports have shown that depletion of certain autophagy-related genes frequently occurs in a variety of cancers to enable proper tumorigenesis (229). The first step in tumorigenesis is the transformation of a pre-malignant cell into a neoplastic precursor, which can be inhibited by autophagy via cellular senescence. Autophagy can remove damaged mitochondria that would otherwise release reactive oxygen species (ROS) or micronuclei that act as a source of genomic instability. Autophagy can also maintain healthy stem cell niches; however, upon completion of malignant transformation, autophagy may promote tumor growth by maintaining cancer stem cells, especially in combination with tumor hypoxia (230).

#### 4.2.2 Tumor promotion

In advanced late-stage tumors, autophagy was shown to promote tumor growth. At this stage, the tumor is exposed to various types of stress such as nutrient deprivation or hypoxia. Due to high proliferation rates of the tumor, the availability of high amounts of nutrients is indispensable. Because of the recycling capacity of autophagy, nutrients can be made available for the tumor to fulfill its high energetic demands. Therefore, the tumor can hijack the autophagic machinery resulting in enhanced stress tolerance and cancer cell survival (229, 231)

RAS-driven cancers are especially dependent on autophagy for tumor progression and malignancy (232). Autophagy inhibition in those tumors affects multiple aspects of cancer metabolism such as lipid metabolism (233), amino acid recycling (234) and nucleotide homeostasis (235), making it a promising therapeutic strategy for RAS-driven cancers.

Furthermore, autophagy has been implicated in chemotherapy resistance. Treatment with chemotherapeutics like 5-fluorouracil or cisplatin was shown to induce protective autophagy in a variety of different cancer types, thereby restricting the efficacy of those drugs and promoting resistance. Combining autophagy inhibitors with chemotherapeutic drugs may provide a promising strategy for overcoming cancer drug resistance (236–239).

A lot of effort has been poured into finding suitable biomarkers associated with autophagy that could guide available treatments options. LC3B has been identified as a potential biomarker and high LC3B rates can predict poor prognosis in hepatocellular carcinoma as well as colon cancer and TNBC, while p62 has also been used as a biomarker for colon cancer (240–242). In TNBCs with high LC3B rates, autophagy inhibition showed promising results as proof of concept (243).

Since many pancreatic cancers seem to rely on high autophagy rates for tumor growth, autophagy inhibitors may prove beneficial for the treatment of pancreatic tumors (244, 245). Autophagy inhibition may sensitize pancreatic cancer to radiotherapy, as radiation was shown to induce autophagy (246). In pancreatic cancer, autophagy has been linked with the degradation of MHC-I, thereby limiting antigen presentation and promoting immune evasion (247).

Currently the only two Food and Drug Agency (FDA)-approved autophagy inhibitor drugs are chloroquine (CQ) and hydroxychloroquine (HCQ). In the context of cancer, multiple clinical trials using CQ or HCQ either in combination or as monotherapy are currently in progress showing partly promising results in terms of anticancer effects and toxicity in early phase studies. Since the majority of clinical trials are either in phase I or II, further evaluation and testing is needed regarding long term toxicity and potential adverse effects associated with those drugs (248–252). Unfortunately, one of the main downsides of using CQ and HCQ is the high degree of off target effects (253). An effort to produce highly specific small molecule inhibitors that might be better suited as targeted treatments in patients is under way (18, 254).

In conclusion, targeting autophagy might be a promising strategy for cancer therapy. However, the lack of clinically suitable autophagy inhibitors and the accompanying effects on the tumor, which are dependent on the genetic makeup and stage of the tumor, must be considered and further improved to ensure favorable outcomes for patients.

### 4.3 Autophagy and hypoxia

Cancer cells are part of the tumor microenvironment (TME), which includes non-cancerous cells present in the tumor as well as surrounding stroma and various cytokines and chemokines that are secreted. The TME generally tends to promote cancer progression as well as therapy resistance (255, 256). One of the players shaping the TME and its tumorigenic effects is hypoxia. Due to their fast proliferation, tumors can rapidly outgrow their vasculature leading to low oxygen levels. To compensate for their lack of oxygen, cells utilize hypoxic signaling mediated mainly by HIF1 (257). HIF1 is a transcription factor that can bind to hypoxia response elements in promoters, which leads to the transcription of genes involved in cell survival, proliferation and autophagy (258–260). HIF1 is a heterodimer that consists of HIF1α and HIF1β. Both subunits are constitutively expressed, however, during normoxia HIF1α is polyubiquitinated by the VHL complex mediating its degradation by the proteasome. Upon induction of hypoxia, HIF1α is no longer polyubiquitinated and can form a functional heterodimer with HIF1β, allowing it to translocate to the nucleus and induce transcription (14, 261).

BCL2-interacting protein 3 (BINP3) and BCL2-interacting protein 3-like (BINP3L) are induced by HIF1. Under normoxia, B-cell lymphoma 2 (Bcl-2) forms low-affinity complexes with Beclin-1, thereby decreasing the overall rate of autophagy. Under low oxygen, BINP3 and BNIP3L are upregulated, bind to Bcl-2 in place of Beclin-1, freeing it from the complex and leading to higher rates of autophagy (262).

Another way by which hypoxia can lead to the induction of autophagy is via ER stress followed by the unfolded protein response (UPR). Prolonged hypoxia leads to ER stress, which can be sensed by protein kinase R-like endoplasmatic reticulum kinase (PERK) (263). PERK induces the translation of ATF4, which induces the expression of the transcription factor C/EBP homologous protein (CHOP) (264). CHOP regulates the expression of autophagy genes and the switching between autophagy and apoptosis (265).

Since severe hypoxia also goes hand in hand with lack of nutrients, AMPK, which is one of the main regulators of metabolic stress, is often activated under hypoxia and can regulate autophagy directly by phosphorylation of ULK1 as well as indirectly by inactivation of mTOR (266).

### 4.4 Interaction of cGAS-STING signaling with autophagy in cancer

Given that cGAS-STING signaling is intricately connected with autophagy, the following section will provide insights into how autophagy affects cGAS-STING signaling in the context of cancer.

In breast cancer cell lines, irradiation-induced Bcl-2-associated X (BAX)-dependent permeabilization of the mitochondrial membranes results in the release of mtDNA into the cytosol, thus increasing cGAS-STING dependent type I IFN production. Autophagy negatively regulates this effect by clearing cytosolic dsDNA and thereby promoting radio-resistance. Yamazaki et al. further identified cytosolic DNA originating from mitochondria as the main driver of the abscopal response, a phenomenon whereby distant tumor sites are affected by irradiation of the main malignant lesion. In autophagy-deficient mice, irradiation shows a strong induction of type I interferons, which are responsible for the regression of abscopal tumors. The authors linked this to clinical outcomes in breast cancer patients; autophagy rates inversely correlate with the amount of mtDNA and type I IFN response and patients with higher amounts of mtDNA show improved disease-free survival and overall survival (205).

In esophageal squamous cell carcinoma (ESCC), however, disruption of mitochondrial biogenesis through transcription factor A, mitochondrial (TFAM) downregulation strongly induces autophagy and promotes ESCC cancer growth, in line with the poor overall survival rate of ESCC patients with low expression of TFAM. Mechanistically, TFAM downregulation promotes the release of mtDNA, thereby promoting non-canonical autophagy through the activation of the cGAS-STING pathway. In TFAM-deficient ESCC cells, ISGs are significantly downregulated supporting the idea that STING degradation through autophagy attenuates the expression of type I IFN genes, thereby promoting cancer cell progression (267). Blockage of autophagy using the late-stage autophagy inhibitor bafilomycin A1 was shown to reduce STING degradation leading to enhanced downstream signaling and improved anti-tumor response (268).

Further building on the inverse relationship between autophagy and cGAS-STING signaling, inhibition of autophagy has been shown to increase the cGAS-STING signaling activity by promoting the accumulation of irradiation-induced dsDNA in the cytosol. Moreover, inhibition of autophagy combined with irradiation induces a potent antitumor effect in PD-L1-deficient lung cancer mouse models via cGAS-STING-mediated T-cell activation, suggesting a possible combination therapy of irradiation, anti-PD-L1 immune checkpoint blockade and autophagy inhibition in patients with lung cancer (218).

Furthermore, inhibition of the deubiquitinase TRAF-binding domain-containing protein (TRABID) that regulates cell division leads to accumulation of micronuclei through defects in cell division and autophagy. Moreover, cGAS is no longer autophagically degraded and can recognize micronuclei generated by mitotic defects, thereby inducing cGAS-STING signaling (269).

In addition to the induction of type I IFNs via phosphorylated IRF3, the activated cGAS-STING signaling cascade also induces the NF-κB signaling pathway, leading to the production of inflammatory cytokines like IL-6 or TNF-α, chemokines such as CXCL10 and cell cycle regulators (104, 270–272). cGAS-STING-dependent IL-6 production contributes to the survival of chromosomally unstable cancers in an IL-6-STAT3 dependent manner, at the same time inhibiting STAT1 signaling, preventing apoptosis and promoting cell survival and growth (226). In addition to cell survival and proliferation, STAT3 has been linked to various other protumorigenic functions such as angiogenesis and immune escape and is often categorized as immunosuppressive (273, 274). Inhibition of hypoxia-induced autophagy was shown to disrupt pSTAT3 signaling through accumulation of p62, which selectively induces pSTAT3 degradation by the proteasome system and thereby restores cytotoxic T-cell mediated killing of tumor cells (273). In triple negative breast cancer cell lines, myc overexpression was shown to downregulate STING signaling and its downstream effectors in a STAT3-dependent manner (274).

In colorectal cancer, IL-6 induces Beclin-1 phosphorylation at Y333 by the JAK2 kinase, which increases Beclin-1 affinity for VPS34. This enhances autophagic rates and promotes chemoresistance in a STAT3-independent manner. Patients with Beclin-1 Y333 phosphorylation also display increased chemotherapy resistance and poor prognosis (275). Recently, it was shown that IL-6-induced autophagy can also contribute to cancer cachexia, a condition characterized by severe loss of body weight and increased muscle wasting and is associated with an increased risk of cancer treatment failure and poor overall survival. Mechanistically, cancer cells capable of inducing cancer cachexia are secreting IL-6, which subsequently induces autophagy in differentiated muscle cells via IL-6 signaling and therefore might be relevant for the characteristic muscle and weight loss during cancer cachexia (276).

### 4.5 Clinical implications

#### 4.5.1 Sting agonists

Given the complex involvement of STING signaling in the cancer immunity cycle and its capability to induce an anti-tumor immune response, efforts have been made to develop new STING agonists. In a pre-clinical setting, numerous STING agonists such as DMXAA or ADU-S100 have shown promising results by inducing a potent anti-tumor immune response either alone or in combination with other conventional cancer therapies including radio- and chemotherapy (277–281). Therefore, it was only a matter of time until STING agonists entered clinical trials, with DMXAA being the first. Contrary to pre-clinical studies where DMXAA was shown to induce a robust anti-tumor immune response, clinical trials did not show any beneficial effects (282, 283). Later studies, however, revealed that DMXAA binds to mouse STING, but not to its human counterpart, explaining the lack of response in clinical trials (284). The new STING agonist ADU-S100 capable of activating human STING showed modest efficacy with low overall response rates (285, 286). Nevertheless, multiple clinical trials testing different cyclic dinucleotides [e.g., BMS-986301 (NCT03956680) or BI 1387446 (NCT04147234)] either alone or in combination with other cancer therapies are currently ongoing and will ultimately determine the future relevance of STING agonists in a clinical setting (www.clinicaltrials.gov). One of the major constraints is the delivery of these drugs, which significantly limits their clinical application. This led to the development of new delivery systems such as extracellular vesicles loaded with cyclic dinucleotides (referred to as exoSTING) or SYNB1891, an engineered *E. coli* strain capable of producing cyclic dinucleotides when injected intratumorally (287–289).

While first-generation STING agonists showed only modest activities, new potent STING agonists in combination with other cancer therapies and improved delivery methods might improve treatment options for cancer patients.

#### 4.5.2 Autophagy modulators

Chloroquine and hydroxychloroquine remain the most clinically tested autophagy inhibitors to date, due to their previous FDA approval as anti-malaria drugs. While they appear to be relatively safe, the outcomes of clinical studies are inconsistent, largely due to their lack of specificity (290, 291). This instigated the development of specific small molecule inhibitors of autophagy such as SAR405 or repurposing of FDA approved drugs (254, 292–297). The chemoproteomic platform has also been used to screen for novel small molecule inhibitors targeting autophagy and could provide an invaluable tool in the future (298–300). Despite promising preclinical results with new inhibitors, CQ or HCQ are still predominantly used in clinical trials in combination with histone deacetylase inhibitors, antiangiogenic kinase inhibitors or MEK inhibitors in different tumors (301–304).

In recent years, activation of autophagy has also been considered for cancer therapy (296, 305). For example, fasting cycles, especially in combination with chemotherapeutic agents, led to increased long-term survival in mice (306). Starvation, as induced by caloric restriction, downregulates mTOR activity and activates autophagy (307, 308). Diets or drugs mimicking caloric restriction such as hydroxycitrate could effectively delay progression in breast cancer, melanoma and lung cancer and led to the autophagy-dependent depletion of regulatory T cells in mice (307, 309).

Considering the heterogenicity of the tumor and its microenvironment, modulation of autophagy should be assessed on a case-by-case basis depending on specific tumor types and biomarkers to avoid adverse effects (19, 291, 310–312). For example, chronic inhibition of autophagy is associated with an irreversible increase in cancer risk (310). In breast cancer, samples positive for both LC3B and nuclear HMGB1 positively correlate with prolonged metastasis-free survival (313, 314). Conversely, LC3-positive extracellular vesicles (EVs) can promote lung metastasis in breast cancer patients and the number of LC3-positive EVs positively correlates with disease progression (315). In comparison, high autophagy rates correlate with poor survival, lymph node metastasis and hepatic metastasis in gastric cancer (316). Low LACTB expression and high LC3 levels are associated with poor patient responses to neoadjuvant chemotherapy (317). However, in multiple myeloma, patients with strong immunoreactivity to Beclin-1 or LC3 show significantly improved overall survival compared to those with medium or low levels of Beclin-1 or LC3 (318). This heterogenicity of tumors and the resulting convoluted nature of biomarkers makes personalized cancer treatments challenging, especially considering that combinations of multiple markers correlate with different outcomes compared to single markers.

#### 4.5.3 Radiotherapy

Radiotherapy is a commonly used treatment approach for many different types of cancer with approximately 50% of all cancer patients receiving radiotherapy during the course of their illness. It is used to treat both benign and malignant cancers, alone or in combination with other therapeutical approaches such as chemotherapy or surgery (319).

Radiotherapy can activate the immune system through the cGAS-STING signaling pathway and thereby potentiate immunotherapy with immune checkpoint inhibitors (ICIs) (209, 320, 321). Irradiated tumor cells secrete type I interferons, which activate several immune cell types, including dendritic cells and T cells (320). Indeed, in several preclinical studies with immunocompetent mouse models radiotherapy was shown to synergize with ICIs (322–325). Clinical evidence is, however, conflicting. A combinatorial treatment of radiotherapy with ICIs was effective in lung, prostate cancer and melanoma but failed to show superior effects over radiotherapy or immunotherapy alone in glioblastoma and head and neck cancer (326–331).

Conventionally, the main goal of radiotherapy has been to kill cancer cells, notwithstanding the local immunosuppression through various pathways such as vascular disruption and hypoxia, which might result in diminished recruitment of certain immune effector cells, exhaustion of effector T cells, and stimulation of regulatory T cells (321, 332–334). Furthermore, standard radiotherapeutic approaches sometimes target the local draining lymph nodes to reduce the risk of tumor recurrence. Draining lymph nodes, however, are essential for T-cell priming, immunostimulatory effects and successful checkpoint blockade (321, 335). Therefore, efforts must be made to adapt radiotherapy to work in a more immunostimulatory way and thereby enable a clinically superior radiotherapy-immunotherapy combinatorial approach. For example, hypofractionated radiation was shown to be more immunogenic compared to a single high dose in breast cancer and was shown to sensitize mice with breast cancer, melanoma, colon carcinoma or pancreatic cancer to ICI (336–340). Synergistic effects of hypofractionated radiation and ICI were confirmed in patients with lung cancer or melanoma (328, 330). Spacing fractions apart in personalized ultrafractionated stereotactic adaptive radiotherapy (PULSAR) has shown promising results in combination with ICI in colon and lung cancer mouse models, which is currently being evaluated in clinical studies (341). Moreover, particle therapy with protons or carbon ions was shown to induce a stronger immunogenic response compared to conventional photon therapy in mouse melanoma models (342).

## 5 Conclusion and outlook

Autophagy and cGAS-STING signaling are two highly connected pathways. Both are crucial for defense against pathogen invasion and different diseases such as cancer or neurodegeneration. Here we provided a comprehensive overview of the current state of knowledge on how these two pathways interact. cGAS-STING signaling is tightly regulated by autophagy at almost all levels. In the context of cancer therapy, targeting cGAS-STING signaling is a promising strategy to activate the innate immune system to fight and ultimately kill tumor cells. In most cases, autophagy negatively regulates cGAS-STING signaling and may therefore negatively impact cancer therapy. Preclinical data indicates that boosting cGAS-STING signaling while inhibiting autophagy might be a promising treatment option to improve patients’ outcomes. Enhancing the immunostimulatory effects of radiotherapy, improving delivery methods for STING agonists and developing specific autophagy modulators will allow further investigation of their clinical potential as monotherapy or combinatorial therapy. Moreover, approaches such as proteogenomics (343–346) that provide information about tumor subtypes and tumor microenvironment may reveal new biomarkers to guide personalized therapy options. Finally, further efforts in understanding cGAS-STING signaling and autophagy pathways individually as well as their interactions will be instrumental for their targeting in cancer.

## Author contributions

MS: Writing – original draft, Writing – review & editing. PF: Writing – original draft, Writing – review & editing. ME: Writing – review & editing, Visualization. JW: Writing – review & editing. SK-G: Writing – review & editing. DS: Writing – review & editing, Writing – original draft.
